# Surround suppression and normalization in a model of coupled balanced cortical networks with short-term synaptic plasticity

**DOI:** 10.1186/1471-2202-16-S1-P249

**Published:** 2015-12-18

**Authors:** Sara Konrad, Tatjana Tchumatchenko

**Affiliations:** 1 Theory of Neural Dynamics, Max-Planck Institute for Brain Research, Frankfurt, 60438, Germany

## Introduction

The input currents to neurons in the cortex are under many circumstances highly variable with a mean intensity considerably below the firing threshold. As a consequence, the spiking activity of cortical neurons is strongly fluctuating such that the coefficient of variation of the inter-spike interval distribution of individual neurons is approximately equal to 1, implying almost Poisson-like spiking. In addition to this characteristic activity, the connectivity of excitatory and inhibitory neurons in cortex is sparse and irregular. It has been shown in models that super-threshold excitatory and strong inhibitory input currents, which nearly cancel for individual neurons, can lead to this irregular spiking activity [1]. However, balanced networks of excitatory and inhibitory neurons are characterized by a strictly linear relation between stimulus strength and network firing rate, making it hard to perform more complex computational tasks like the generation of receptive fields, multiple stable activity states or normalization, which for example has been measured in visual cortex (eg. [2,3]).

Synapses displaying activity dependent short-term plasticity (STP) have been previously reported to give rise to a non-linear network response with potentially multiple stable states for a given stimulus [4].

**Figure 1:**
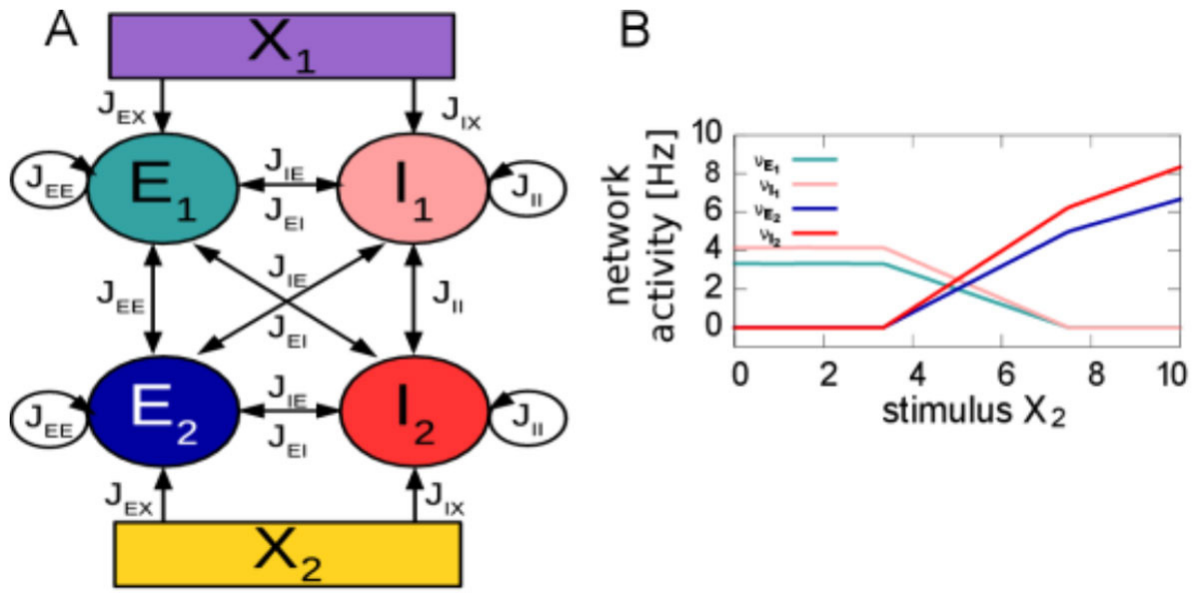
**A. Schematic illustration of the network topology**. E_1 _and I_1 _represent excitatory and inhibitory neuronal populations of network 1, receiving input from an external population X_1_. Same for network 2. Coupling of neuronal populations is indicated by the arrows, synaptic strengths by the J_yz_. **B**. Schematic illustration of the resulting activity in the network for constant stimulus X_1 _and varying stimulus X_2_. In this model we observe the activity of network 1 being suppressed when activity in network 2 increases. Colors of lines represent the population corresponding to panel A.

In this study, we analyze analytically and numerically the computational properties of two interconnected balanced networks, receiving independent stationary stimuli. This situation can be viewed as a simple instantiation of two interconnected cortical columns. For an illustration of the network topology, see Figure 1A. We demonstrate that these stimuli are normalized by the system and that increasing the stimulus to one network, suppresses the activity of the neighboring network (see Figure 1B). Thereby, normalization and suppression are linear in stimulus strength when STP is disabled and becomes non-linear with activity dependent synapses.
